# Homœopathic Medical Society of New York

**Published:** 1884-04

**Authors:** J. L. Moffat

**Affiliations:** Secretary


### HOMŒOPATHIC MEDICAL SOCIETY OF THE STATE OF NEW YORK.

At the thirty-third annual meeting of the Homœopathic Medical Society of the State of New York, February 12th and 13th last, the following officers were elected:

*President*—Edward S. Coburn, M. D., 91 Fourth Street, Troy.

*Vice-Presidents*—Henry C. Houghton, M. D., 12 West Thirty-ninth Street, New York; H. M. Dayfoot, M. D., Rochester; A. P. Hollett, M. D., Havana.

*Secretary*—John L. Moffatt, M. D., 17 Schermerhorn Street, Brooklyn.

*Treasurer*—H. L. Waldo, M. D., West Troy.

CENSORS: *Northern District*—Drs. A. W. Holden, Glens Falls; W. T. Laird, Watertown, and D. E. Southwick, Ogdensburg.

*Southern District*—S. P. Burdick and F. E. Doughty, New York, and H. Minton, Brooklyn.

*Middle District*—N. B. Covert, Geneva; M. O. Terry, Utica, and W. E. Milbank, Albany.

*Western District*—F. Park Lewis, Buffalo; Asa S. Couch, Fredonia, and T. D. Spencer.

The next semi-annual meeting will be held at Binghamton, September 9th and 10th, 1884, and the annual meeting at Albany on the second Tuesday and Wednesday of February, 1885. A cordial invitation is extended to all friends of Homœopathy.

